# Cold Storage Exacerbates Renal and Mitochondrial Dysfunction Following Transplantation

**Published:** 2016-02-27

**Authors:** S Shrum, LA MacMillan-Crow, N Parajuli

**Affiliations:** Department of Pharmacology and Toxicology, University of Arkansas for Medical Sciences, Little Rock, AR, USA

**Keywords:** Kidney, Mitochondria, Cold storage, Transplantation

## Abstract

Long-term renal function is compromised in patients receiving deceased donor kidneys which require cold storage exposure prior to transplantation. It is well established that extended cold storage induces renal damage and several labs, including our own, have demonstrated renal mitochondrial damage after cold storage alone. However, to our knowledge, few studies have assessed renal and mitochondrial function after transplantation of rat kidneys exposed to short-term (4 hr) cold storage compared to transplant without cold storage (autotransplantation). Our data reveal that cold storage plus transplantation exacerbated renal and mitochondrial dysfunction when compared to autotransplantation alone.

## Introduction

Kidney transplantation is the preferred modality of treatment for end stage kidney disease. While advances in tissue-type matching and immunosuppressive protocols have greatly reduced short-term graft dysfunction after renal transplantation, long-term graft function continues to be problematic, especially in patients receiving deceased donor kidneys [1–3]. The kidneys of deceased donors are routinely flushed and stored in cold storage (CS) solutions [4]. Short-term CS lowers the metabolic demand, yet, prolonged CS causes extensive tissue damage and is associated with a higher incidence of delayed graft function and poor long-term outcome after transplant [1,5,6]. Despite this, the mechanism of how CS negatively impacts overall graft function and survival is not well understood. Many laboratories, including our own, have published that renal CS (*in vitro* and *ex vivo*) induces mitochondrial injury, reactive oxygen species (ROS) generation, and cell death [7–12]. However, characterization of mitochondrial function after renal CS plus transplantation remains unknown. The respiratory enzyme complexes (complexes I-V) comprise the electron transfer chain, and through mitochondrial respiration generate ATP as the terminal product with ROS as a byproduct. When mitochondrial dysfunction occurs, cells are deprived of ATP and ROS are increased, consequently impacting numerous cellular pathways responsible for tissue regeneration/repair or damage. Therefore, our goal was to evaluate the contribution of short-term CS to early renal dysfunction and altered mitochondrial respiratory function following CS plus transplantation (CS/Tx) by comparing to autotransplant (ATx) (without CS exposure), and sham surgery.

## Methods

### Animals

Male Lewis rats weighing 200 – 250 g were used as transplant donors and recipients in this study. All of the animal protocols were approved by the Institutional Animal Care and Use Committee (IACUC) at the University of Arkansas for Medical Sciences (UAMS), and all animal experiments described below were performed in compliance with the IACUC at UAMS using NIH guidelines.

### Syngeneic rat renal transplant model with cold storage

Orthotopic renal transplant surgery was performed in male Lewis rats. For the donor rat surgery, rats were anesthetized using isoflourane, and the left kidney was flushed with cold saline solution containing heparin (100 units/ml) via the aorta. The left kidney was removed *en bloc* (with the renal artery, vein and ureter attached) and stored in University of Wisconsin (UW) solution at 4°C for 4 hr. For the recipient rat surgery, rats were anesthetized using isoflourane, the native left kidney was removed, and the donor left kidney was transplanted using end-to-end anastomosis as described previously. The surgical ischemia time was ~ 45 min. The right native kidney was immediately removed, making renal function dependent on the transplanted left kidney. The ureter was anastomosed end-to-end over a 5 mm PE-50 polyethylene stent. Postoperatively, the animals were given 0.9% (w/v) NaCl in the abdominal cavity and placed under a heating lamp to recover from the anesthesia. Rats were given buprenorphine (0.06 mg/kg, SC) for pain. After 24hr of reperfusion, the transplanted left kidney and blood were collected under anesthesia and saved as ***4hr cold storage plus transplantation (4h CS/Tx)*** group (n = 4).

### Control groups

#### Autotransplant surgery

The autotransplant (ATx) surgery was performed as described in the recipient surgery method, except that the left kidney was removed and immediately transplanted in the same rat without CS exposure, followed by right nephrectomy. The surgical ischemia time was ~ 45 min. After 24 hr, the transplanted kidney was harvested under anesthesia, and the organs were referred to as ***Autotransplantation (ATx)*** group (n=4).

#### Sham surgery

Rats underwent identical surgery (right nephrectomy), but without the renal transplantation (Sham operation). The right kidney from a donor rat was removed and the left kidney remained functioning for 24 hr, and then the sham kidney and blood were harvested and saved as the ***Sham*** group (n = 4).

#### Blood chemistry

Blood chemistry was determined in heparinized blood (arterial) using a hand-held clinical chemistry analyzer, iSTAT^™^ [13], and cartridges (CHEM8^+^) as described by the manufacturer (Vetscan^®^, Abaxis, USA).

### High resolution respirometry (HRR)

Mitochondrial respiratory complex activity was measured in the saponin permeabilized renal biopsies by high resolution respirometry (HRR) (Oroboros instruments - Oxygraph-2k, Innsbruck, Austria), according to substrate-inhibitor-titration (SIT) protocol as described earlier [11,14]. DATLAB 4.2 software (Oroboros) was used to analyze data, and tissue respiration was shown as oxygen flux (pmol/mg/s).

### ATP assay

An ATP-luciferase-based bioluminescence assay kit (Sigma, MO, USA) and TD 20/20 luminometer (Turner Designs Sunnyvale, CA, USA) were used to measure ATP levels in the sham and transplant kidney tissue lysates as described earlier [14].

### Statistical analysis

Results are presented as mean ± standard error of the mean (S.E.M.) using Graph Pad Prism software (version 4.0). The Student’s *t-*test was used to compare differences between the mean of two groups at a 95% level of confidence. Differences with a *P* value <0.05 were considered statistically significant. Comparisons were made between the groups: sham vs. ATx, sham vs. 4hrCS/Tx, and AutoTx vs. 4hrCS/Tx.

## Results and Discussion

**Figure 1:**
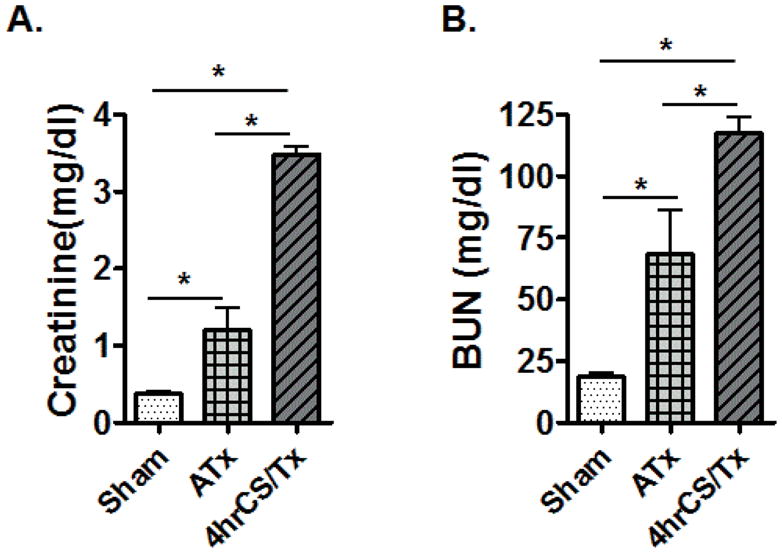
Cold storage worsens renal function after transplantation. Whole blood (arterial) creatinine and blood urea nitrogen (BUN) from the sham, autotransplant (ATx), and 4hCS/Tx rats were analyzed using hand-held clinical chemistry analyzer (iSTAT^™^) and Chem8^+^ cassettes as described in materials and methods. Values were expressed as Mean ± S.E.M. (n = 3–4); * indicates means are significantly different (P < 0.05).

We previously reported in isolated rat and pig kidneys that CS alone induced significant renal and mitochondrial injury [10,11]. In this study, we evaluated renal function after 4 hr CS combined with transplantation using a syngeneic transplantation model to minimize confounding effects of the host immune system. Similarly, the autotransplant model without CS was chosen to evaluate the effects of warm ischemia combined with surgical trauma on kidney and mitochondrial function following transplantation. Sham rats showed no significant changes in plasma creatinine (Cr; ~0.5 mg/dl) and blood urea nitrogen (BUN; ~20 mg/dl), but these parameters were increased after autotransplant (ATx; Figure 1A and 1B). However, rat kidneys exposed to 4 hr CS plus transplantation showed markedly increased levels of Cr and BUN (4hrCS/Tx, Figure 1A and 1B).

Our laboratory previously showed that CS (4 hr) alone induces altered renal mitochondrial function (reduced respiratory complex I-IV activity, using a spectrophotometric assay and isolated renal mitochondria), leading to renal tubular injury [10]. However, it was not clear if mitochondrial damage extended to post-transplant. In the current study, High Resolution Respirometry (HRR) was used, which, in contrast to the spectrophotometric assay, monitors real-time mitochondrial respiration in fresh renal biopsies, thereby excluding the possibility of introducing assay artifacts on functional analysis via mitochondrial isolation.

**Figure 2:**
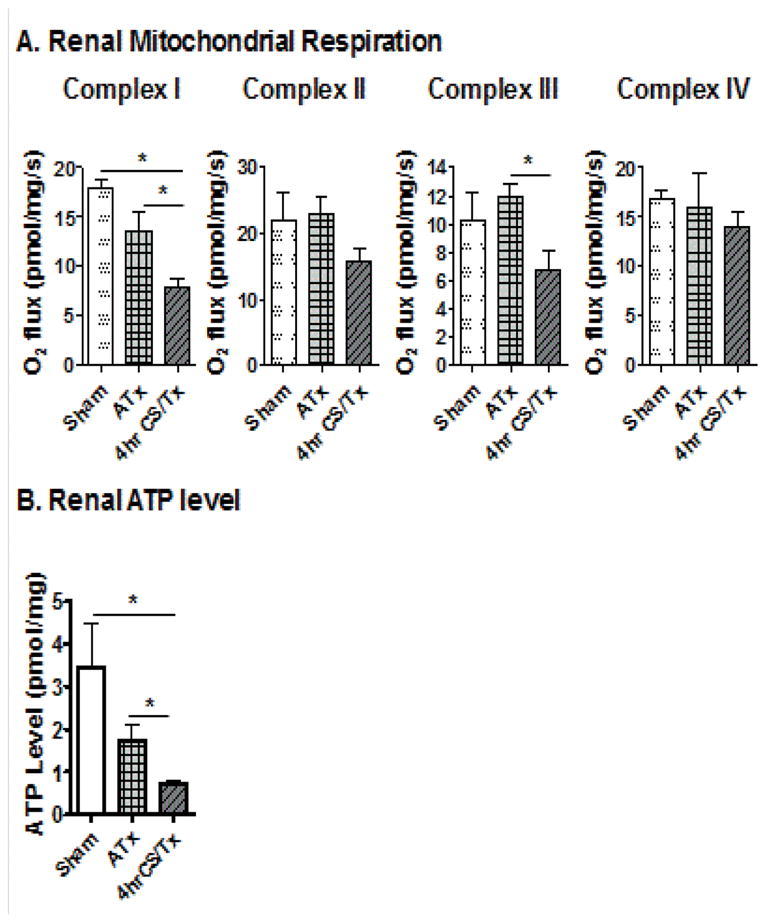
Cold storage worsens mitochondrial function after transplantation. (A) Graph showing respiratory complex I, II, III, and IV activity of the electron transport chain using high resolution respirometry in sham kidneys, or after transplantation with 4 hr cold storage (CS/Tx) or without CS (autotransplant, ATx). (B) Graph showing ATP levels in renal homogenates in sham, CS plus transplantation (CS/Tx), or autotransplant (ATx) groups. Error bar indicates Mean ± S.E.M. (n = 3–4); *indicates means are significantly different (P < 0.05).

A significant decline in renal mitochondrial complex I respiratory function (~50%) was detected by reduced oxygen flux following CS/Tx when compared to sham or ATx groups (Figure 2A). Similarly, complex III activities was significantly declined after CS/Tx when compared to ATx (Figure 2A). There was no change in complex II or IV activity following CS/Tx when compared to sham or ATx. To our knowledge, this is the *first report demonstrating a reduction of renal mitochondrial respiratory complex (I and III) activity following renal CS/Tx*. Since compromised respiratory complex activity can lead to reduced ATP levels, we also evaluated ATP levels. CS plus transplantation showed a dramatic loss of renal ATP when compared to the sham or ATx ATP levels (Figure 2B). These data suggest that short-term (4 hr) CS negatively impacts mitochondrial function following transplantation.

Several other studies have documented renal dysfunction following various times of CS, although many are much longer than 4 hr [4,15,16]. Our study revealed, for the first time, that CS (4 hr) leads to a selective respiratory complex inhibition (I and III, but not II and IV) and ATP depletion following transplantation. Therefore, we anticipate that loss of mitochondrial function is a critical event that leads to energy depletion and exacerbated renal dysfunction during CS/Tx.

The profound decline in complex I activity occurring after CS/Tx is significant because complex I has been shown to be a major source of ROS in many diseases [17–19]. In addition, complex III, which was also inactivated after CS/Tx, also participates in ROS generation [20–22]. Future studies will evaluate CS-induced oxidant generation and its involvement in the mechanism of respiratory complex inactivation following CS/Tx. Similarly, additional studies employing longer CS and reperfusion times are warranted to evaluate how CS negatively impacts mitochondrial function that causes sustained loss of ATP following CS/Tx. One limitation of the current study is that rat kidneys were harvested from living animals, whereas clinically the majority of kidney donors are harvested from deceased patients. Future studies could be performed using a non-heart beating donor rat prior to CS, but the rationale for the current study was to dissect the effect that CS alone has on mitochondrial damage. Another limitation is certainly the time of CS as well as reperfusion, and new studies are underway to determine the extent of mitochondrial/renal damage with longer CS times (up to 24 hr). Finally, new studies will need to look at 3–7 days post-transplant to determine if function is improved or worsened.
